# A systematic mapping review of individual participant factors related to eating disorder risk collected in behavioural weight management trials

**DOI:** 10.1007/s40519-026-01866-0

**Published:** 2026-05-12

**Authors:** Hannah Melville, Hiba Jebeile, Sol Libesman, Sarah P. Garnett, Kylie E. Hunter, Isabelle R. Jardine, Rabia Khalid, Sasha J. Lorien, Jonathan G. Williams, Louise A. Baur, Anna L. Seidler, Natalie B. Lister

**Affiliations:** 1 Children’s Hospital Westmead Clinical School, The University of Sydney, Sydney, NSW Australia; 2 Charles Perkins Centre, The University of Sydney, Sydney, NSW Australia; 3 NHMRC Clinical Trials Centre, Faculty of Medicine and Health, The University of Sydney, Sydney, NSW Australia; 4 German Centre for Child and Adolescent Health, University Medicine Rostock (Universitätsmedizin Rostock), University of Rostock, Rostock, Germany

**Keywords:** Behavioural weight management, Obesity, Eating disorders, Mapping review, Individual participant data

## Abstract

**Purpose:**

The purpose of this study was to identify which factors potentially relevant to eating disorder risk were collected by trials included in the Eating Disorders In weight-related Therapy (EDIT) Collaboration.

**Methods:**

Databases were systematically searched to March 2022, and clinical trial registries to May 2022, to identify behavioural weight management randomised controlled trials assessing eating disorder risk in adolescents (10–18 years) and adults (≥ 19 years) with overweight/obesity. Individual participant data were requested from eligible trials across 11 domains (demographics, anthropometry, medical history, history of dieting and weight loss attempts, mental health, personality, psychosocial health, sleep quality, weight stigma, eating behaviours, cardiometabolic health), including 58 participant factors identified through a consensus study as relevant to eating disorder risk. Trial data were mapped and summarised descriptively.

**Results:**

We identified 106 eligible trials, of which 46 (20 adolescent, 26 adult) individual participant datasets were provided. Adolescent and adult datasets both included factors across a median (range) of 6 (4–9) domains. There was broad coverage across demographics, medical history, anthropometry, mental health, psychosocial health, and eating behaviours, where each domain was included in > 50% of datasets. Each of the remaining domains were included in ≤ 50% of datasets.

**Conclusion:**

Trials collected many diverse factors potentially relevant to eating disorder risk. Further exploration is needed within underrepresented domains, such as weight stigma, to better understand their contribution to eating disorder risk within behavioural weight management.

**Level of evidence:**

Level V, Opinions of respected authorities, based on descriptive studies, narrative reviews, clinical experience, or reports of expert committees.

**Supplementary Information:**

The online version contains supplementary material available at 10.1007/s40519-026-01866-0.

## Introduction

Behavioural weight management is integral within obesity treatment interventions in both adolescents and adults [1–3]. Individuals may present for obesity interventions with undiagnosed eating disorders or disordered eating. A 2025 systematic review estimated that, in adults presenting for obesity treatment, 14% present with binge eating disorder and 26% with self-reported moderate binge eating. [4] Behavioural weight management interventions are commonly used to improve weight outcomes in adolescents [5, 6] and adults [7, 8]. However, there are concerns about some weight management strategies such as dieting and other weight control behaviours, due to their shared risk factors with eating disorders [9–11]. Recent systematic reviews and meta-analyses have found eating disorder risk improves or remains unchanged for most adolescents and adults following weight management interventions [8, 12–16]. A small subset of individuals, however, may experience poor eating disorder outcomes during or following behavioural weight management interventions [14, 15]. Given this, eating disorder development within behavioural weight management is a serious and important safety concern. There is currently an evidence gap in understanding who may develop eating disorders, and how, during weight management interventions.

In 2021, the Eating Disorders In weight-related Therapy (EDIT) Collaboration (hereon referred to as EDIT) was formed to address this evidence gap. This international collaboration uses individual participant data meta-analyses and intervention deconstruction to understand the individual level interaction between behavioural weight management interventions and eating disorder risk. [17, 18] To achieve this, a list of individual participant factors potentially relevant to change in eating disorder risk during behavioural weight management interventions was developed. These factors were based on previous literature and stakeholder consultation [17, 19], and were used to inform the data collection process from trialists.

While eating disorder risk factors are generally well established [20], our previous scoping review found key eating disorder risk factors such as childhood abuse and trauma were not considered in models of eating disorder development for people with obesity [21]. Further, there was a lack of longitudinal evidence on risk factors within a weight management context [21]. In order to comprehensively assess eating disorder risk during obesity treatment and inform future analysis, there is first a need to systematically map [22] availability of factors and highlight areas of research paucity. Therefore, the primary aim of this systematic mapping review was to identify which factors potentially relevant to eating disorder risk were collected by behavioural weight management trials included in the EDIT Collaboration. The secondary aim was to identify critical research gaps to guide future data collection and enable further exploration of eating disorder risk within behavioural weight management interventions.

## Methods

This systematic mapping review was based on a protocol for a systematic review and individual participant data meta-analysis which aims to evaluate individual eating disorder risk during behavioural weight management [17]. The protocol was prospectively registered on PROSPERO (CRD42021265340). A systematic mapping review was selected as this method applies a rigorous approach to identifying and describing evidence and evidence gaps in a broad topic [22]. This mapping review expands the original protocol’s scope to summarise the factors potentially relevant to eating disorder risk collected by eligible behavioural weight management trials. This review follows the Preferred Reporting Items for Systematic Reviews (PRISMA) [23].

### Eligibility criteria

Randomised controlled trials eligible for inclusion in EDIT were identified through a systematic search as previously described [17]. In brief, eligible trials were behavioural weight management interventions aiming to improve a weight-related outcome in either adolescents (10–18 years at baseline) or adults (≥ 19 years at baseline) with overweight or obesity as reported by trialists, or defined as body mass index (BMI) z-score > 1 or BMI ≥ 85th percentile in adolescents, and BMI > 25 kg/m^2^ in adults. Eligible trials were required to collect at least one eating disorder symptom or behaviour using a clinical interview or validated questionnaire at baseline and post-intervention and/or follow-up.

### Information sources

Electronic databases (MEDLINE, Embase, PsycINFO, and Scopus) were searched to March 2022 and clinical trial registries (ClinicalTrials.gov and the WHO International Clinical Trials Registry Platform) to May 2022, with additional handsearching of relevant sources. Eligible trialists were invited by email to join EDIT and share de-identified individual participant data.

### Selection process

Identified studies were uploaded to Covidence [24] for title and abstract and full text screening. Screening was independently conducted in duplicate with conflicts resolved by discussion or a third reviewer. Clinical trial registry records were screened by one reviewer, with uncertainties resolved by a second reviewer [25].

### Identifying individual participant factors

A consensus process was used to identify all individual participant factors (n) potentially relevant to change in eating disorder risk in the context of weight management interventions. The findings of the consensus study have been previously published [19]. In brief, factors were first derived from the literature and through consultation workshops with the EDIT Scientific and Stakeholder Advisory Panels. An international online survey then rated factors for relevance, and captured any relevant factors not already identified. The resulting factors (*n* = 58) deemed potentially relevant to change in eating disorder risk during weight management were collated, and grouped thematically into 11 different domains: demographics (*n* = 14), anthropometry (*n* = 4), medical and family history (*n* = 7), history of dieting and weight loss attempts (*n* = 3), mental health (*n* = 6), personality traits (*n* = 1), psychosocial health (*n* = 4), sleep quality (*n* = 1), weight stigma (*n* = 3), eating behaviours (*n* = 10), and cardiometabolic health (*n* = 5). As specified in the eligibility criteria, all included trials collected data using validated eating disorder measures.

### Data collection process

For each of the 58 participant factors, data were requested from eligible trials who agreed to join EDIT. Data from each trial were mapped against the EDIT variable list. For each variable, the details of measurement and reporting were recorded, e.g. name of questionnaire and scoring. This process was completed independently by one researcher and checked by a second. Trialists were contacted to query any unclear, incomplete, or missing information and details were updated accordingly.

### Synthesis methods

A narrative summary of studies was conducted according to the Synthesis Without Meta-analysis (SWIM) guidelines [26]. Factors were summarised using descriptive statistics including counts and percentages. To visualise the co-occurrence of collected domains, UpSet graphs were developed with R version 4.4.2 (2024-10-31) [27] using the UpSetR [28] statistical package.

## Results

**Fig. 1:**
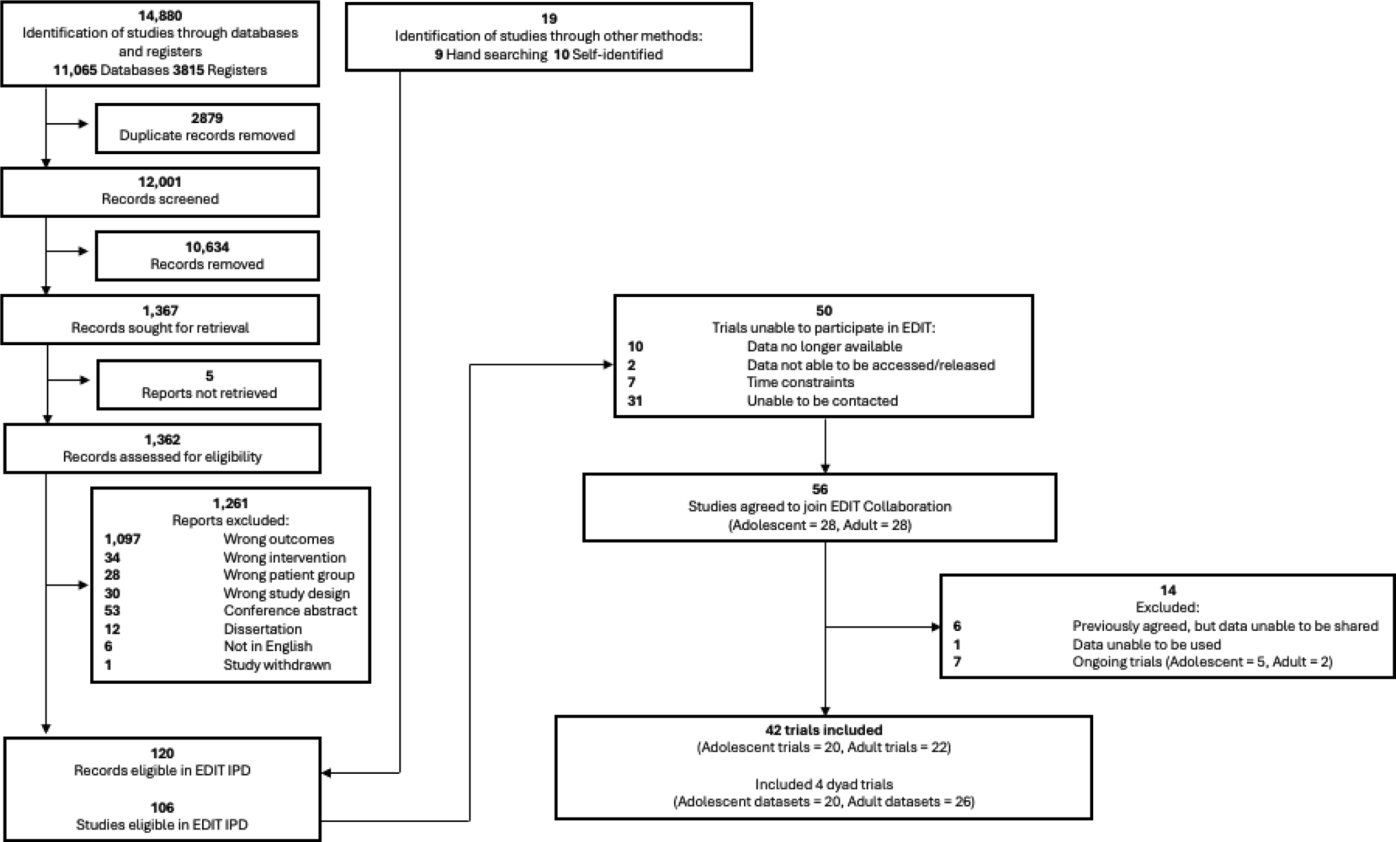
PRISMA flow diagram

From 14,880 records screened, 106 trials were eligible and invited to join EDIT. From these, 56 trials agreed to join EDIT and 42 (20) adolescent [29–59] and 22 adult [60–99] trials) shared individual participant data. Four adolescent trials included parent/caregiver dyad data which were then integrated with other adult trials to form 26 adult datasets (k) in total (Fig. 1). Most trials (*n*) were conducted in United States (*n* = 23), followed by Australia (*n* = 7) and the United Kingdom (*n* = 3). Trials were completed in a variety of settings including university (*n* = 16), hospital outpatient (*n* = 10), and primary care (*n* = 4). See Supplementary Table 1 for summarised trial information including participant enrolment dates, and the individual participant factors collected per trial.

### Eating disorder symptoms and behaviours

Consistent with the eligibility criteria, all datasets provided data on eating disorder symptoms or behaviours. In adolescents, most datasets provided data on global eating disorder-related symptoms (*k* = 16), followed by eating disorder behaviours or episodes (*k* = 9), and binge eating-related symptoms (*k* = 4). In adult datasets, binge eating-related symptoms (*k* = 14) were most commonly provided, followed by global eating disorder-related symptoms (*k* = 13), and eating disorder behaviours or episodes (*k* = 12). Further information on eating disorder symptoms and behaviours is provided in Table 1.

**Table 1:** Number of collected and provided eating disorder symptoms and behaviours including descriptions, questionnaires, or tools, by domain for adolescent and adult datasets

Eating disorder symptoms or behaviours	Description Validated questionnaires or assessment tools	Number of adolescent datasets (total = 20)	Number of adult datasets (total = 26)
Eating disorder diagnosis	Clinical diagnosis/assessment of any eating disorder including binge eating disorder, anorexia nervosa, bulimia nervosa, and other specified feeding or eating disorder (OSFED) (not by validated questionnaire)	2	6
Global eating disorder-related symptoms	Overall presence of global eating disorder-related symptoms, including attitudes, cognitions, and/or behaviours Eating Disorder Examination (EDE), Eating Disorder Examination Questionnaire (EDE-Q), Eating Attitudes Test (EAT), Kids Eating Disorder Survey (KEDS), and youth versions	16	13
Binge eating-related symptoms	Binge eating symptoms and behaviours (continuous scores) Binge Eating Scale (BES), Multidimensional Assessment of Eating Disorder Symptoms (MAEDS) binge eating subscale, Bulimic Investigation Test of Edinburgh (BITE)	4	14
Presence/absence of binge eating	Participants self-report binge eating Eating Disorder Diagnostic Scale (EDDS), Binge Eating Disorder Screener (BEDS)	2	2
Eating disorder behaviours or episodes	Purging, laxative misuse, diuretic misuse, excessive exercise, loss of control and binge eating symptoms and episodes Eating Disorder Examination (EDE), Eating Disorder Examination Questionnaire (EDE-Q), Loss of Control Eating Scale (LOC-ED), Questionnaire on Eating and Weight (QEWP), and youth versions	9	12

### Factors and domains potentially related to eating disorder risk

Of the 11 domains, both adolescent and adult datasets included factors across a median (range) of six (4–9) domains. See Table 2 for the summary of all available adolescent and adult individual participant factors with descriptions, showing the number of total datasets that included each factor. The domains of demographics, medical and family history, anthropometry, mental health, psychosocial health, and eating behaviours were included by > 50% of datasets. Some domains were underrepresented, meaning factors within a domain were less frequently measured, in both adolescents and adults. The domains included by ≤ 50% of datasets were history of dieting and weight loss attempts, personality traits, sleep quality, weight stigma, and cardiometabolic health. Tabulated results for adolescent and adult datasets with median (range) of included factors per dataset for each domain are presented in Supplementary Table 2.

**Table 2:** Number of collected and provided factors, including descriptions, questionnaires or tools, by domain for adolescent and adult datasets

Factor	Description Questionnaires or assessment tools	Number of adolescent datasets (total = 20)	Number of adult datasets (total = 26)
Demographics			
Age	Age at the time of enrolment	20	26
Sex/gender	Sex/gender—defined as either sex at birth or self-report gender	20	26
Race/ethnicity	Self-report race or ethnicity of participant (or parents if not available for adolescent)	16	20
Language status	Primary language spoken at home, language proficiency	4	5
Family structure	Household number of adults, household number of children, household total number of people	12	5
Household income	Total household income, equivalised household income	7	10
Partner status	Partner status of adult participant or parent/carer of adolescent	10	17
Education status	Highest education level of the adult participant or of the parent/carer of the adolescent participant	16	19
Employment status	Employment status of the adult participant or of the parent/carer of the adolescent participant	11	13
Socio-economic status	Socio-economic index of area/position Hollingshead index, Barratt Simplified Measure of Social Status	9	5
Food insecurity	Family history of or current food insecurity	0	0
Health literacy	Health literacy of the participant	0	0
Parenting	Parenting style, feeding style/practices (*adolescent trials only)*	3	N/A
Support	General social or peer support (*adolescent trials only), h*ealth behaviour specific social or peer support Medical Outcomes Study Social Support Survey, Social Support for Eating/Exercise Habits Survey, Network Support for Healthy Behaviours Interview	8	7
Anthropometry			
Weight	Weight (kg)	20	25
Height	Height (cm)	20	25
Waist circumference	Waist circumference (cm)	8	13
Adiposity	Body fat percentage Dual-energy X-ray absorptiometry (DEXA), Bioelectrical Impedance Analysis (BIA), air-displacement plethysmography (BODPOD)	10	13
Medical and family history			
Puberty or menopause	Puberty stage (*adolescent trials only),* puberty timing (*adolescent trials only)*, age at onset of menopause (*adult trials only)*	4	2
Medical history	History of type 2 diabetes mellitus, type 1 diabetes mellitus, bariatric surgery, cardiometabolic disease, mental illness, other relevant medical history such as obstructive sleep apnoea *(adult trials only)*	13	19
Eating disorder history	History of eating disorders, history of eating disorder treatment	5	15
Family medical history	Family history of obesity, diet-related chronic disease, type 2 diabetes mellitus, bariatric surgery, eating disorders	11	2
Medication history	Current medication history (any medication), weight-related medication history	10	21
Age of obesity onset	Age of onset of obesity	1	1
Life events which may impact upon body weight	Prior life events which may impact upon body weight e.g. pregnancy, smoking cessation	1	3
History of dieting and weight loss attempts			
Prior weight loss attempts	Number of prior weight loss attempts, total weight lost achieved	3	6
Type of prior weight loss attempts	Prior weight loss attempt using diet, weight loss pharmacotherapy, other method, or method unclear	6	6
Dieting duration	Any previous dieting *(adolescent trials only),* number of dieting attempts, age of onset of dieting	6	2
Mental health			
Mental health diagnoses	Diagnosed by clinical interview/assessment/medical history (not by validated questionnaire)	1	7
History of trauma	History of trauma	0	2
Addiction, substance/smoking use	Addiction (general) *(adolescent trials only)*, alcohol abuse *(adult trials only)*, smoking status *(adult trials only)*, drug abuse *(adult trials only)* Clinical interview, Alcohol and Health Questionnaire	4	12
Self-harm and suicidality	Self-harm, suicide attempt/ideation Beck Depression Inventory (BDI), Patient Health Questionnaire-9 (PHQ-9), Suicidality Assessment, Children’s Depression Inventory, Centre for Epidemiologic Studies Depression Scale Revised-10	2	3
Depression symptoms	Symptoms of depression Children’s Depression Inventory (CDI, CDI-2), Child Behaviour Check List (CBCL)2014withdrawn/depressed subscale or internalising score (where no withdrawn/depressed subscale available), Centre for Epidemiologic Studies Depression Scale (CESDR-10), Depression, Anxiety and Stress Scale (DASS, DASS-21)—depression subscale, The Reynolds Adolescent Depression Scale (RADS), Short Mood and Feelings Questionnaire (SMFW), Youth Self-Report (YSR)—internalising score, Beck Depression Inventory (BDI, BDI-II), Patient Health Questionnaire (PHQ-2, PHQ-9), Hospital Anxiety and Depression Scale—depression subscale, Multiaxial Assessment of Eating Disorder Symptoms (MAEDS)—depression subscale, Brief Symptom Inventory—depression subscale, The Symptom Checklist-90 (SCL90)—depression subscale	19	19
Anxiety symptoms	Symptoms of anxiety Child Behaviour Check List (CBCL)—anxious/depressed subscale, State-Trait Anxiety Inventory for Children (STAIC), Depression, Anxiety and Stress Scale (DASS, DASS-21)—anxiety subscale, Youth Self-Report (YSR)—anxious/depressed subscale or internalising scale (where no anxious/depressed subscale available), Short Scale Anxiety Sensitivity Index (ASI-3), Screen for Child Anxiety Related Emotional Disorders (SCARED), General Anxiety Disorder (GAD-7), Hospital Anxiety and Depression Scale—anxiety subscale, Beck Anxiety Inventory (BAI), Brief Symptom Inventory—anxiety subscale, The Symptom Checklist-90 (SCL90)—anxiety subscale	11	15
Personality traits			
Personality traits	Personality traits Ten-Item Personality Inventory (TIPI), Big Five Inventory (44 items)	0	2
Psychosocial health			
Self-esteem	Self-esteem Self-Perception Profile for Children/Adolescents (SPPC/SPPA), Harter Self-Perception Profile for Adults (SPPA)—global self-worth subscale, Rosenberg Self-Esteem Scale (RSE)	8	3
Body dissatisfaction	Body dissatisfaction Self-Perception Profile for Children/Adolescents (SPPC/SPPA)—physical appearance subscale, Body Appreciation Scale (BAS), Body Esteem Scale for Adolescents and Adults, Body Shape Questionnaire (BSQ), Body Uneasiness Test-A (BUT-A), Body Morphe Assessment 2.0	10	5
Quality of life	Quality of life Pediatric Quality of Life Inventory (PedsQL), RAND Health Survey (SF-36, SF-12, SF-36 with only vitality, bodily pain, mental and general health subscales), RAND 12-Item health survey, The World Health Organization Quality of Life Brief Version (WHOQOL-BREF), The Impact of Weight on Quality of Life-Lite (IWQOL-Lite), The Impact of Weight on Quality of Life-Kids (IWQOL-Kids), Quality of Life Enjoyment and Satisfaction Questionnaire	8	8
Stress	Stress Depression, Anxiety and Stress Scale (DASS, DASS-21)—stress subscale, The Perceived Stress Scale (PSS, different versions used), Cohen Perceived Stress Scale	6	11
Sleep quality			
Sleep quality	Sleep quality Questionnaires include: The Pittsburgh Sleep Quality Index (PSQI), Women's Health Initiative Insomnia Rating Scale (WHIIRS)	2	6
Weight stigma			
Weight bias	Weight bias internalisation Weight Bias Internalisation Scale	2	0
Weight-related bullying	Weight-related bullying Perceived weight discrimination scale (adapted from the Everyday Discrimination Scale), Perception of Teasing Scale (POTS), Appearance Teasing Inventory (ATI)	3	0
Weight talk	Weight talk (peer or family)	0	0
Eating behaviours			
Dietary restraint	Dietary restraint measured using validated eating behaviour measures Dutch Eating Behaviour Questionnaire (DEBQ)—restraint subscale, Three Factor Eating Questionnaire (TFEQ, TFEQ-R21, TFEQ-R18)—dietary restraint subscale, Eating Inventory—cognitive restraint subscale	5	14
Disinhibition	Disinhibition related to eating Three Factor Eating Questionnaire (TFEQ, TFEQ-R21 or TFEQ-R18)—disinhibition/uncontrolled eating subscale, Mindful Eating Questionnaire—disinhibition subscale, Eating Inventory—disinhibition subscale	1	11
Impulsivity	Impulsivity related to eating Child Eating Behaviour Questionnaire (CEBQ)—responsiveness to food subscale, UPPS-P Impulsive Behavior Scale—negative urgency scale, Short form of the UPPS-P Impulsive Behavior Scale (S-UPPS-P)	3	2
Emotional eating	Emotional eating Emotional Eating Scale for Children (EES-C), Dutch Eating Behaviour Questionnaire—emotional eating subscale, Child Eating Behavior Questionnaire (CEBQ)—emotional over- and under-eating subscales, Three Factor Eating Questionnaire (TFEQ-R21, TFEQ-R18)—emotional eating subscale	8	6
Self-efficacy towards eating	Self-efficacy towards eating Child Dietary Self-Efficacy Scale (CDSS), Eating Self-Efficacy Scale (ESES), Weight Efficacy Lifestyle Questionnaire (WEL), The Eating/Exercise Habits Confidence Survey (EHCS)	2	4
Night eating	Night eating	0	0
Grazing	Grazing Repetitive Eating Questionnaire (Rep(eat)-Q), The Grazing Questionnaire	1	2
External eating	External eating/ food responsiveness Eating in Absence of Hunger (EAH)—external eating subscale, Dutch Eating Behaviour Questionnaire (DEBQ)—external eating subscale	5	7
Secret eating	Secret eating	0	0
Food rules	Food rules, narrow range/limited food choices or acceptability Multiaxial Assessment of Eating Disorder Symptoms (MAEDS)—avoidance of forbidden foods subscale, Child Behaviour Checklist Questionnaire (CEBQ)—fussiness subscale	2	3
Cardiometabolic health			
Blood pressure	Systolic blood pressure, diastolic blood pressure	6	12
Blood lipids	Total cholesterol, high-density lipoprotein cholesterol, triglycerides	6	11
Insulin and glucose	Insulin and glucose	6	11
C-reactive protein	C-reactive protein	5	8
Liver enzymes	Aspartate aminotransferase (AST), alanine aminotransferase (ALT), gamma-glutamyl transferase (GGT)	6	5

**Fig. 2:**
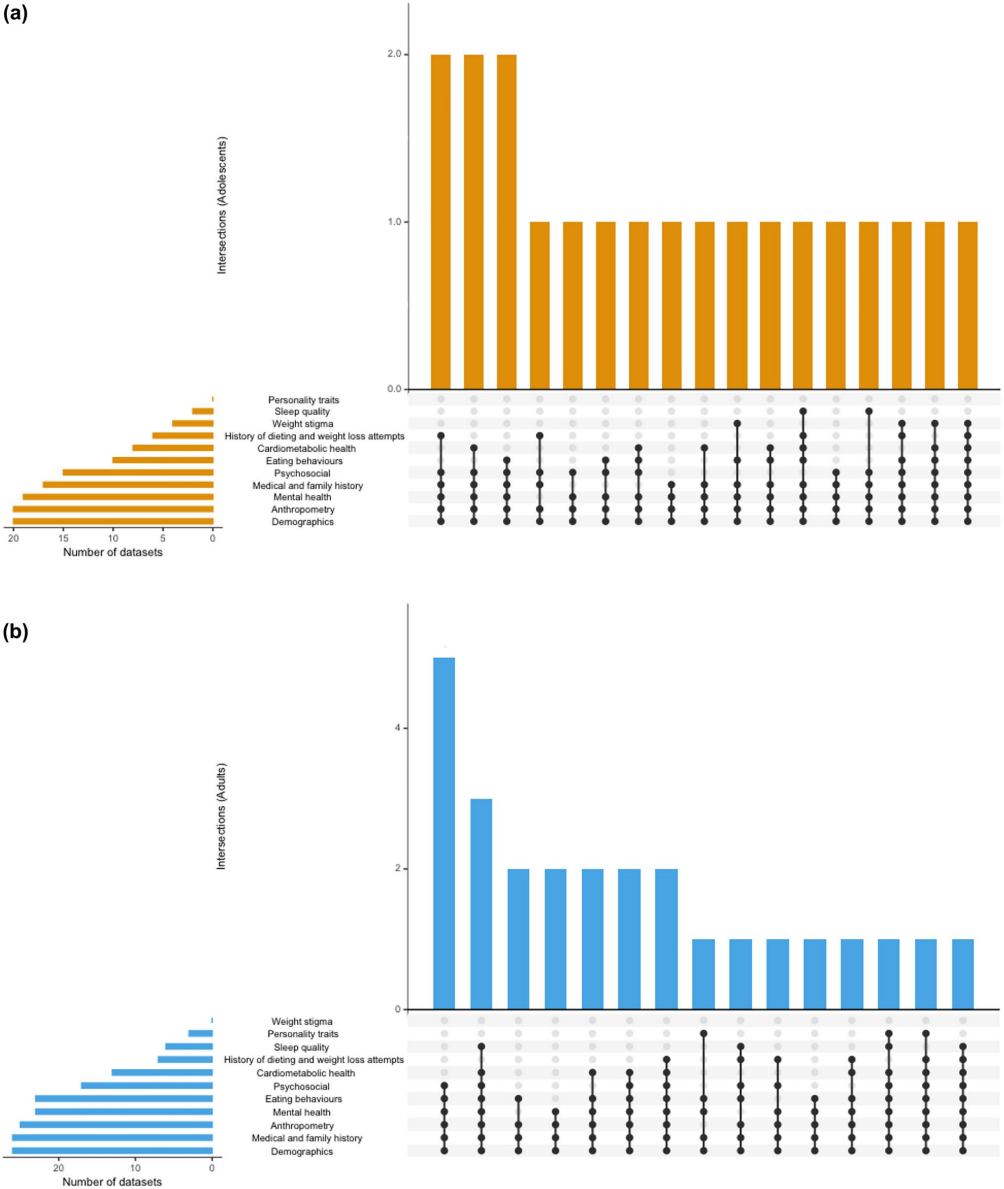
**a** Frequency of combination of domains for adolescent datasets. The lower panel lists the domains of individual participant factors included in 20 adolescent datasets. The horizontal bar chart (bottom left) shows the number of datasets including each domain. The connected black dots (bottom right) represent the combination of domains within datasets. The vertical bar chart shows the number of datasets with each combination of domains represented by the connected black dots. i.e. the combination of demographics, anthropometry, mental health, medical and family history, psychosocial and history of dieting, and weight loss attempt domains is shown in the first bar on the vertical bar chart and was included in two datasets. **b** Frequency of combination of domains for adult datasets. The lower panel lists the domains of individual participant factors included in 26 adult datasets. The horizontal bar chart (bottom left) shows the number of datasets including each domain. The connected black dots (bottom right) represent the combination of domains within datasets. The vertical bar chart shows the number of datasets with each combination of domains represented by the connected black dots, i.e. the combination of demographics, medical and family history, anthropometry, mental health, eating behaviours, and psychosocial domains is shown in the first bar on the vertical bar chart and was included in five datasets

Across the 20 adolescent datasets, there were 17 unique combinations of domains reported, as shown in Fig. 2a. Across the 26 adult datasets, there were 15 unique combinations of domains reported, as shown in Fig. 2b. One domain combination was found across five adult datasets [69, 74, 87, 91, 99]. Domains included in this combination were demographics, medical and family history, anthropometry, mental health, eating behaviours, and psychosocial health.

### Demographics

All adolescent (*k* = 20/20) and adult (*k* = 26/26) datasets included factors within the demographics domain. Out of 14 factors, the median number (range) of factors per dataset was 7 (2–10) in adolescents and 6 (2–9) in adults. All adolescent (*k* = 20/20) and adult (*k* = 26/26) datasets included sex at birth or self-reported gender. Across adolescents and adults, datasets included various measures of social determinants of health such as education status (*k* = 16/20; *k* = 19/26), household income (*k* = 7/20; *k* = 10/26), support (*k* = 8/20; *k* = 7/26), and socio-economic status (*k* = 9/20; *k* = 5/26).

### Anthropometry

All adolescent (*k* = 20/20) and most adult (*k* = 25/26) datasets included factors within the anthropometry domain. Out of four factors, the median number of factors per dataset was 3 (2–4) in adolescents and 3 (2–4) in adults. Weight and height were included by all 20 adolescent datasets and half (*k* = 10/20) reported adiposity, e.g. body fat percentage. Similarly, in adults most (*k* = 25/26) datasets included weight and height, and half included adiposity (*k* = 13/26).

### Medical and family history

Most adolescent (*k* = 17/20) and all adult (*k* = 26/26) datasets included factors within the medical and family history domain. Out of seven factors, the median number of factors per dataset was 3 (1–5) for adolescents and 2 (1–5) for adults. In adolescents, the most frequently included factors were medical history (*k* = 13/20), family medical history (*k* = 11/20), and medication history (*k* = 10/20). In adult datasets, the most commonly included factors were medication history (*k* = 21/26), medical history (*k* = 19/26), and eating disorder history (*k* = 15/26). Few included data on life events which may influence body weight (adolescent *k* = 1/20; adult *k* = 3/26) and age of obesity onset (adolescent *k* = 1/20; adult *k* = 1/26).

### History of dieting and weight loss attempts

Less than half of adolescent (*k* = 6/20) and adult (*k* = 7/26) datasets included factors on history of dieting and previous weight loss attempts. Out of three factors, the median number of factors per dataset was 3 (2–3) in adolescents and 2 (1–3) for adult datasets. Few adolescent trials (*k* = 3/20) included data on prior weight loss attempts, e.g. dieting or pharmacotherapy, and few adult trials (*k* = 2/26) included data on dieting duration.

### Mental health

Almost all adolescent (*k* = 19/20) and adult (*k* = 23/26) datasets included factors within the mental health domain. Out of six factors, the median number of factors per dataset was 2 (1–4) in adolescents and 2 (1–5) in adults. Depression (adolescent *k* = 19/20; adult *k* = 19/26) and anxiety symptoms (*k* = 11/20; *k* = 15/26) were most frequently included. Other aspects of mental health were less frequently included, for example, history of trauma (adolescent *k* = 0/20; adults *k* = 2/26), self-harm and suicidality (adolescents *k* = 2/20; adults *k* = 3/26), and mental health diagnoses, e.g. depression or anxiety (adolescents *k* = 1/20; adults *k* = 7/26).

### Personality traits

Three adult dataset (*k* = 3/26) assessed personality traits, the single factor, with no data available for adolescents.

### Psychosocial health

Most adolescent (*k* = 15/20) and adult (*k* = 17/26) datasets included factors within the psychosocial health domain. Out of four factors, the median number of factors per dataset was 2 (1–3) in adolescents and 1 (1–4) in adults. Within adolescents, body dissatisfaction was the most frequently included factor (*k* = 10/20), followed by quality of life (*k* = 8/20). Within adult datasets, stress was the most commonly included factor (*k* = 11/26), followed by quality of life (*k* = 8/26).

### Sleep quality

Few adolescent (*k* = 2/20) and adult (*k* = 6/26) datasets included sleep quality, the single factor in this domain.

### Weight stigma

Few adolescent datasets (*k* = 4/20) included factors related to the experience of weight stigma. Out of three factors, the median number of factors per dataset was 1 (1–2). No data were available for adults.

### Eating behaviours

Half of adolescent (*k* = 10/20) and most adult (*k* = 23/26) datasets included factors within the eating behaviours domain. Out of ten factors, the median number of factors per dataset was 3 (1–4) for adolescents and 2 (1–5) for adults. In adolescents, emotional eating was the most frequently included factor (*k* = 8/20), followed by external eating (*k* = 5/20) and dietary restraint when assessed with an eating behaviour measure (rather than an eating disorder measure) (*k* = 5/20). In adults, dietary restraint was the most included factor (*k* = 14/26), followed by disinhibition related to eating (*k* = 11/26) and external eating (*k* = 7/26).

### Cardiometabolic health

Less than half of adolescent (*k* = 8/20) and half of adult (*k* = 13/26) datasets included factors in the cardiometabolic health domain. Out of five factors, the median number of factors per dataset was 5 (1–5) for adolescents and 4 (1–5) for adults. The most frequently included factors in adolescents and adults were blood pressure (*k* = 6/20; *k* = 12/26) and blood lipids (*k* = 6/20; *k* = 11/26).

## Discussion

This review mapped the available individual participant factors relevant to eating disorder risk, as identified in the EDIT consensus study, across 42 behavioural weight management trials [17, 19]. A key strength and finding from this review was that all trials collected factors across multiple and unique combinations of domains, demonstrating widespread coverage of physical and psychosocial health. Some domains, however, were underrepresented in both adolescents and adults, including history of dieting and weight loss attempts, and the experience of weight stigma. The results from this mapping review identified strengths of the current evidence base, as well as research gaps which need to be addressed to fully understand eating disorder risk within behavioural weight management.

Almost all trials collected and provided data on anthropometry, which was expected, given BMI or overweight/obesity status were eligibility criteria. Our findings demonstrate that EDIT trials commonly collected and provided data on mental health, psychosocial health, and eating behaviours. This is likely also a reflection of EDIT’s eligibility criteria, which required reporting of eating disorder outcomes. There were many unique combinations of domains collected by trials. However, the domains of demographics, anthropometry, medical and family history, mental health, eating behaviours, and psychosocial health were the most common combination in both adolescents and adults. Our previous scoping review of eating disorder development pathways in people with obesity identified some risk factors were infrequently considered, particularly in adolescents. These included dietary restraint, loss of control eating, emotional eating, body dissatisfaction, and self-esteem [21]. In contrast, this mapping review found domains representing these factors were measured in more than 50% of trials, suggesting consideration of holistic health beyond anthropometric measures. Importantly, this highlights data availability for future examination of the potential role of these factors when considering eating disorder development pathways in the context of weight management interventions.

Although many trials in this review were found to collect data across mental health, psychosocial health, and eating behaviours, concerns have been raised that these outcomes are not routinely and consistently assessed in obesity clinical trials [100–102]. Previous systematic reviews have reported the effect of behavioural weight management on these outcomes and emphasised the need to measure and report them [100, 102–108]. It is suggested that lack of resources, expertise, and capacity may be contributing to the paucity of data [101]. Further, when these outcomes are measured, there is significant inconsistency in their reporting and the assessment tools used [100–102, 109]. Such variability impacts the reliability of evidence synthesis, thereby reducing statistical power for meta-analyses. For example, a 2021 meta-analysis in adults reported insufficient evidence on the impact of behavioural weight interventions on select outcomes at intervention end [102]. This, along with infrequent outcome measurement across obesity clinical trials, indicates there are ongoing challenges to evidence synthesis which limit contribution to the evidence base.

This review highlighted some underrepresented domains in behavioural weight management trials. One such domain is weight stigma, which was identified in the EDIT consensus study [19] as an important factor to consider in our understanding of eating disorder risk. A 2025 scoping review of 46 studies identified weight stigma as a common construct within eating disorder development pathways [21]. We speculate this research gap may be due to multiple reasons. Firstly, there is no clear guidance for trialists on which aspect of weight stigma to measure, e.g. internalised weight stigma or experience of weight stigma from the environment, and how to assess these experiences. A 2021 systematic review reported on 18 different weight stigma measures, finding most focused on internalised weight stigma, and were graded as very low or low quality [110]. The measurement of weight stigma, both internalised and experienced, is important as its impact on health is largely missing within the literature [111]. Further research is needed to explore the role of weight stigma in relation to eating disorder risk, as well as on health outcomes more broadly. Secondly, weight stigma may be considered or addressed in a trial without a specific measurement. This was demonstrated in EDIT adolescent trials which specifically included strategies to address weight stigma such as education to increase resilience to weight stigma [112]. Further, efforts to address weight stigma may not always be protocolised or documented, but part of routine clinical care. For example, using person-first language and having access to appropriate medical equipment are good clinical practice but rarely documented [113].

This review revealed few trials collected data related to the history of dieting and weight loss attempts. Previous dieting attempts have been identified as a potential early indicator of eating disorder risk [114]. For example, a 5-year longitudinal study in adolescents (*n* = 2516) found dieting with the intention to lose weight was significantly associated with binge eating in both females and males [115]. However, a 2016 review highlighted the ongoing difficulty in defining dieting history and a weight loss attempt, and suggested dietary restriction can be associated with both positive and negative outcomes during behavioural weight management interventions, depending on the individual [116]. To our knowledge, there are limited validated assessment tools to report previous dieting history and weight loss attempts [117]. The Weight and Lifestyle Inventory has been used to assess dieting and weight loss history in adults within clinical practice; however, it has not been formally validated [118]. Another tool is the Making Weight Inventory which assesses extreme weight control behaviours used to meet a weight requirement, although this tool has only been validated within the military population and refers to behaviours linked to eating disorder pathology such as purging and fasting [119]. Taken together, unclear definitions coupled with limited assessment tools have created barriers in ongoing research in this area.

## Strengths and limits

This mapping review used a systematic approach which enabled the identification of key strengths and gaps within the current evidence base. The results provide a broad overview of the factors collected by behavioural weight management trials within EDIT, which included information beyond primary outcomes as well as unpublished data. There were also limitations. EDIT trials included in this review may not be representative of all behavioural weight management interventions as they have assessed eating disorder risk at multiple time points. Thus, EDIT trials were designed by investigators cognisant of mental health, and this may have further influenced domains included in trials. Trials collected data using a variety of different assessment tools, standardised and more consistent measures may facilitate future evidence synthesis. Further, trials in this review were all conducted in upper-middle- or high-income countries, meaning results may not be generalisable to all countries. This may also indicate some wider health research disparities that should be addressed in future studies. Finally, this mapping review only described included factors from EDIT trials and does not infer factor importance or priority when considering eating disorder risk in behavioural weight management.

## What is already known on this subject?

Eating disorder development during behavioural weight management is an important safety consideration. Yet, factors potentially related to eating disorder risk collected by trials have not been described.

## What this study adds?

All identified trials collected factors across multiple and unique combinations of domains, demonstrating widespread coverage of physical and psychosocial health. Some key areas important to our understanding of eating disorder risk were underrepresented such as weight stigma and history of dieting and weight loss attempts. This mapping review provides insight into future research priorities within eating disorder risk and obesity.

## Conclusion

This systematic mapping review found EDIT trials collected diverse individual factors across many domains potentially relevant to eating disorder risk, with over half providing data across mental health, psychosocial health, and eating behaviours. Some key areas that are important to our understanding of eating disorder risk were underrepresented such as weight stigma and history of dieting and previous weight loss attempts. Importantly, the data availability outlined in this review will inform future analysis and understanding of the potential role of risk factors within eating disorder development pathways in weight management interventions.

## Supplementary Information

Below is the link to the electronic supplementary material.

Supplementary file1 (DOCX 75 kb)

## Data Availability

The authors confirm that the data supporting the findings of this study are available within the article and in online supplementary file. Further enquiries can be directed to the corresponding author.
