# Supplementary material for: A systematic mapping review of individual participant factors related to eating disorder risk collected in behavioural weight management trials
Source: Eat Weight Disord. 2026 May 12;31(1):66. doi: 10.1007/s40519-026-01866-0 (PMC13333562; doi:10.1007/s40519-026-01866-0)
Supplement: Supplementary file 1 — Supplementary file1 (DOCX 75 kb) [file 40519_2026_1866_MOESM1_ESM.docx]

**Supplementary Table 1: Trial characteristics**

| **Author/s, year; Country; Study setting(s); Enrolment start to enrolment end (month/ year)** | **Individual participant factors collected and provided to EDIT** | | | | | | | | | | |
| --- | --- | --- | --- | --- | --- | --- | --- | --- | --- | --- | --- |
|  | **Demographics** | **Medical and family history** | **History of dieting and weight loss attempts** | **Anthropometry** | **Mental health** | **Personality traits** | **Psychosocial health** | **Sleep quality** | **Weight stigma** | **Eating behaviour** | **Cardiometabolic health** |
| **Adolescent trials** | | | | | | | | | | | |
| Bean et al., 2022 (1); Raynor et al., 2021 (2); USA; Primary care; 2016 – 2018 | Age  Sex/gender  Race/ethnicity  Household structure  Household income  Education status | Medical history  Eating disorder history  Medication history | Nil | Weight  Height | Self-harm and suicide history  Depression symptoms | Nil | Nil | Nil | Nil | Nil | Blood pressure |
| Bonham et al., 2017 (3); Dordevic et al., 2015 (4); Australia; Commercial provider; March 2013 – March 2014 | Age  Sex/gender  Race/ethnicity  Language status  Household structure  Partner status  Education status  Employment status  Socioeconomic status  Support | Puberty and menopause  Medical history  Family medical history | Prior weight loss attempts  Type of prior weight loss attempts  Dieting duration | Weight  Height | Nil | Nil | Self-esteem  Body dissatisfaction  Quality of life | Nil | Nil | Nil | Nil |
| Braet et al., 2004 (5); Belgium; Hospital inpatient; September 1996 – September 1999 | Age  Sex/gender | Medical history | Nil | Weight  Height | Depression symptoms  Anxiety symptoms | Nil | Self-esteem  Body dissatisfaction | Nil | Nil | Dietary restraint  Emotional eating  External eating | Nil |
| Croker et al., 2012 (6); UK; Hospital outpatient; January 2004 – June 2007 | Age  Sex/gender  Race/ethnicity  Household structure  Partner status  Education status  Employment status  Parenting feeding and style | Puberty and menopause  Medical history  Family medical history | Nil | Weight  Height  Waist circumference  Adiposity | Depression symptoms | Nil | Self-esteem  Body dissatisfaction  Quality of life | Nil | Weight-based bullying | Dietary restraint  Impulsivity  Emotional eating  Food rules | Blood pressure  Blood lipids  C-reactive protein  Insulin and glucose  Liver enzymes |
| Doyle et al., 2008 (7); USA; Virtual & University; November 2003 – May 2005 | Age  Sex/gender  Race/ethnicity  Household structure  Partner status  Education status | Medication history | Nil | Weight  Height | Depression symptoms  Anxiety symptoms | Nil | Body dissatisfaction  Quality of life  Stress | Nil | Nil | Nil | Nil |
| Jelalian et al., 2006 (8); USA; Hospital outpatient; January 2000 – January 2002 | Age  Sex/gender  Race/ethnicity  Education status  Employment status  Socioeconomic status  Support | Medication history | Nil | Weight  Height | Depression symptoms | Nil | Self-esteem  Body dissatisfaction | Nil | Nil | Nil | Nil |
| Jelalian et al., 2020 (9); Darling et al., 2021 (10); USA; Community;  November 2014 – October 2016 | Age  Sex/gender  Race/ethnicity  Household structure  Partner status  Education status  Employment status  Socioeconomic status | Medical history  Eating disorder history  Family medical history  Medication history | Prior weight loss attempts  Type of prior weight loss attempts  Dieting duration | Weight  Height  Waist circumference | Depression symptoms | Nil | Self-esteem  Body dissatisfaction | Nil | Nil | Nil | Nil |
| Lister et al., 2024 (11); Jebeile et al., 2024 (12); Lister et al., 2020 (13); Australia; Hospital outpatient & virtual; 2018 – 2022 | Age  Sex/gender  Race/ethnicity  Language status  Household structure  Household income  Partner status  Education status  Employment status  Socioeconomic status | Medical history  Family medical history  Medication history | Type of prior weight loss attempts  Dieting duration | Weight  Height  Waist circumference  Adiposity | Depression symptoms  Anxiety symptoms | Nil | Self-esteem  Body dissatisfaction  Stress | Sleep quality | Weight bias | Dietary restraint  Emotional eating  External eating | Blood pressure  Blood lipids  C-reactive protein  Insulin and glucose  Liver enzymes |
| Lofrano-Prado et al., 2022 (14); Brazil; School; March 2018 | Age  Sex/gender | Nil | Nil | Weight  Height  Waist circumference  Adiposity | Addiction/substance abuse  Depression symptoms | Nil | Body dissatisfaction  Quality of life | Nil | Nil | Nil | Nil |
| Mehlenbeck et al., 2009 (15); Jelalian et al., 2010 (16); USA; Hospital outpatient; August 2003 – May 2006 | Age  Sex/gender  Race/ethnicity  Household structure  Household income  Education status  Employment status  Support | Family medical history | Prior weight loss attempts  Type of prior weight loss attempts  Dieting duration | Weight  Height  Waist circumference | Depression symptoms | Nil | Self-esteem  Body dissatisfaction | Nil | Nil | Nil | Nil |
| Newsome et al., 2023 (17); USA; Virtual; October 2019 – January 2021 | Age  Sex/gender  Race/ethnicity  Household structure  Household income  Education status  Support | Medical history  Life events which may impact upon body weight | Type of prior weight loss attempts  Dieting duration | Weight  Height | Depression symptoms | Nil | Quality of life  Stress | Nil | Weight bias  Weight-based bullying | Dietary restraint  Disinhibition  Emotional eating | Nil |
| Ramalho et al., 2020 (18); Portugal; Hospital outpatient & virtual; October 2015 – December 2017 | Age  Sex/gender  Household structure  Partner status  Education status  Employment status | Nil | Nil | Weight  Height  Adiposity | Depression symptoms  Anxiety symptoms | Nil | Quality of life  Stress | Nil | Nil | Impulsivity  Grazing | Blood pressure |
| Skjåkødegård et al., 2022 (19); Skjåkødegård et al., 2016 (20); Norway; Hospital outpatient; February 2014 – October 2018 | Age  Sex/gender  Parenting feeding and style | Nil | Nil | Weight  Height | Depression symptoms  Anxiety symptoms | Nil | Self-esteem  Body dissatisfaction | Nil | Nil | Dietary restraint  Emotional eating  External eating | Nil |
| Vermeiren et al., 2021 (21); Naets et al., 2018 (22); Belgium; Hospital inpatient & outpatient; July 2017 – January 2020 | Age  Sex/gender  Race/ethnicity | Puberty and menopause | Nil | Weight  Height  Waist circumference  Adiposity | Depression symptoms  Anxiety symptoms | Nil | Nil | Nil | Nil | Emotional eating  External eating | Blood pressure  Blood lipids  C-reactive protein  Insulin and glucose  Liver enzymes |
| Vidmar et al., 2021 (23); Vidmar et al., 2020 (24); USA; Hospital outpatient & virtual; NR | Age  Sex/gender  Race/ethnicity  Language status  Household structure  Household income  Partner status  Education status  Employment status | Medical history  Eating disorder history  Family medical history  Medication history | Nil | Weight  Height  Adiposity | Depression symptoms | Nil | Quality of life  Stress | Sleep quality | Nil | Nil | Blood lipids  C-reactive protein  Insulin and glucose  Liver enzymes |
| Vidmar et al., 2023 (25); Vidmar et al., 2019 (26); USA; Hospital outpatient & virtual; January 2019 – July 2021 | Age  Sex/gender  Race/ethnicity  Language status  Household structure  Household income  Partner status  Education status  Employment status | Medical history  Family medical history  Medication history | Nil | Weight  Height | Depression symptoms | Nil | Stress | Nil | Nil | Nil | Blood lipids  Insulin and glucose  Liver enzymes |
| **Dyad trials (trials provided individual participant data for both adolescents and adults, unless otherwise indicated)** | | | | | | | | | | | |
| Epstein et al., 2001 (27); USA; Community; NR | Age  Sex/gender  Race/Ethnicity  Education status  Socioeconomic status  Support | Medical history  Family medical history (adolescents only)  Medication history (adults only) | Nil | Weight  Height  Adiposity | Depression symptoms  Anxiety symptoms | Nil | Nil | Nil | Nil | Nil | Nil |
| Shomaker et al., 2017 (28); USA; Hospital outpatient; September 2012 – July 2014 | Age  Sex/gender  Race/ethnicity  Household structure  Education status  Employment status  Socioeconomic status  Support (adolescents only) | Puberty and menopause (adolescents only)  Medical history  Medication history  Age of obesity onset | Type of prior weight loss attempts  Dieting duration | Weight  Height  Waist circumference  Adiposity (adolescents only) | Mental health diagnoses (adolescents only)  Addiction/substance abuse  Depression symptoms (adolescents only)  Anxiety symptoms (adolescents only) | Nil | Nil | Nil | Nil | Emotional eating (adolescents only)  External eating | Blood pressure  Blood lipids  C-reactive protein  Insulin and glucose  Liver enzymes |
| Wilfley et al., 2007 (29); Goldschmidt et al., 2014 (30); USA; University; NR | Age  Sex/gender  Race/ethnicity  Household income  Partner status  Education status  Employment status  Socioeconomic status  Support | Medical history (adolescents only)  Eating disorder history  Family medical history (adolescents only)  Medication history | Nil | Weight  Height | Addiction/substance abuse (adolescents only)  Depression symptoms  Anxiety symptoms | Nil | Nil | Nil | Weight-based bullying (adolescents only) | Self-efficacy | Nil |
| Wilfley et al., 2017 (31); USA; University; January 2010 – September 2010 | Age  Sex/gender  Race/ethnicity  Household income  Partner status  Education status  Socioeconomic status  Parenting feeding and style (adolescents only)  Support | Medical history (adolescents only)  Eating disorder history  Family medical history (adolescents only)  Medication history | Nil | Weight  Height  Waist circumference (adolescents only)  Adiposity (adolescents only) | Addiction/substance abuse (adolescents only)  Self-harm and suicide history  Depression symptoms  Anxiety symptoms | Nil | Quality of life (adolescents only) | Nil | Nil | Impulsivity (adolescents only)  Emotional eating (adolescents only)  Self-efficacy (adolescents only)  Food rules (adolescents only) | Nil |
| **Adult trials** | | | | | | | | | | | |
| Barnes et al., 2014 (32); Barnes et al., 2017 (33); USA; Primary care & virtual; March 2012 – November 2012 | Age  Sex/gender  Race/ethnicity  Partner status  Education status  Employment status | Medical history  Eating disorder history | Nil | Weight  Height | Mental health diagnoses  History of trauma  Addiction/substance abuse  Depression symptoms | Nil | Nil | Nil | Nil | Self-efficacy | Blood pressure  Blood lipids  Insulin and glucose |
| Beaulieu et al., 2020 (34); UK; University; March 2018 – December 2018 | Age  Sex/gender | Eating disorder history  Medication history | Nil | Weight  Height  Waist circumference  Adiposity | Addiction, substance/smoking use | Nil | Quality of life  Stress | Sleep quality | Nil | Dietary restraint  Disinhibition | Blood pressure |
| Boutelle et al., 2022 (35); Boutelle et al., 2023 (36); Boutelle et al., 2019 (37); USA; University; December 2015 – November 2017 | Age  Sex/gender  Race/ethnicity  Household structure  Household income  Partner status  Employment status  Socioeconomic status  Support | Puberty and menopause  Medical history  Eating disorder history  Medication history | Nil | Weight  Height  Adiposity | Mental health diagnoses  History of trauma  Self-harm and suicide history  Addiction/substance abuse Depression symptoms  Anxiety symptoms | Nil | Stress | Nil | Nil | Dietary restraint  Impulsivity  External eating | Nil |
| Carpenter et al., 2019 (38); USA; Virtual; August 2014 – June 2015 | Age  Sex/gender  Race/ethnicity  Partner status  Education status | Medical history Eating disorder history  Medication history | Nil | Weight  Height | Anxiety symptoms | Nil | Stress | Nil | Nil | Disinhibition | Nil |
| Dalle Grave et al., 2013 (39); Italy; Hospital inpatient; September 2007 – January 2011 | Age  Sex/gender  Race/ethnicity  Language status  Partner status  Education status  Employment status | Medication history | Prior weight loss attempts  Type of prior weight loss attempts | Weight  Height  Waist circumference | Mental health diagnoses  Depression symptoms  Anxiety symptoms | Nil | Body dissatisfaction | Nil | Nil | Dietary restraint  Disinhibition | Blood pressure  Blood lipids  C-reactive protein  Insulin and glucose  Liver enzymes |
| Dassen et al., 2018 (40); Netherlands; Virtual; February 2016 – October 2016 | Age  Sex/gender  Language status  Education status | Medical history  Eating disorder history | Nil | Weight  Height | Nil | Nil | Nil | Nil | Nil | Dietary restraint  Emotional eating  External eating | Nil |
| Eichen 2021 (41); USA; University & virtual; NR | Age  Sex/gender  Race/ethnicity  Household income  Partner status  Education status | Medical history  Eating disorder history  Medication history | Nil | Weight  Height | Addiction/substance abuse  Self-harm and suicide history  Depression symptoms  Anxiety symptoms | Nil | Quality of life  Stress | Nil | Nil | Impulsivity  External eating | Nil |
| Griffin et al., 2013 (42); Cheng et al., 2014 (43); Australia; Hospital outpatient; March 2006 – July 2009 | Age  Sex/gender  Race/ethnicity  Language status  Household structure  Employment status | Medical history  Eating disorder history  Family medical history  Medication history  Life events which may impact upon body weight | Prior weight loss attempts  Type of prior weight loss attempts | Weight  Height  Waist circumference  Adiposity | Mental health diagnoses | Nil | Self-esteem | Nil | Nil | Dietary restraint  Disinhibition | Blood pressure  Blood lipids  C-reactive protein  Insulin and glucose  Liver enzymes |
| Imayama et al., 2011 (44); Mason et al., 2019 (45); USA; Primary care; 2005 – 2008 | Age  Sex/gender  Race/ethnicity  Partner status  Education status  Employment status  Support | Puberty and menopause  Medical history  Family medical history  Medication history  Life events which may impact upon body weight | Prior weight loss attempts  Type of prior weight loss attempts | Weight  Height  Waist circumference  Adiposity | Addiction/substance abuse  Depression symptoms  Anxiety symptoms | Nil | Stress | Sleep quality | Nil | Dietary restraint  Disinhibition  Emotional eating | Blood pressure  Blood lipids  C-reactive protein  Insulin and glucose |
| Jospe et al., 2017 (46); Jospe et al., 2018 (47); Taylor et al., 2015 (48); New Zealand; University; November 2014 – April 2015 | Age  Sex/gender  Race/ethnicity  Household structure  Household income  Partner status  Education status  Employment status | Medical history  Medication history | Prior weight loss attempts  Type of prior weight loss attempts  Dieting duration | Weight  Height  Waist circumference  Adiposity | Nil | Personality traits | Stress | Nil | Nil | Dietary restraint  Emotional eating  External eating | Blood pressure  Blood lipids  C-reactive protein  Insulin and glucose |
| Lin et al., 2023 (49); USA; University;  January 2021 – September 2021 | Age  Sex/gender  Race/ethnicity | Medical history  Medication history | Nil | Weight  Height  Waist circumference  Adiposity | Addiction/substance abuse  Depression symptoms | Nil | Quality of life | Sleep quality | Nil | Food rules | Blood pressure  Blood lipids  Insulin and glucose |
| Martin et al., 2019 (50); Myers et al., 2014 (51); USA; University;  November 2010 – March 2015 | Age  Sex/gender  Race/ethnicity  Household income  Partner status  Education status  Employment status | Medical history | Nil | Weight  Height  Waist circumference  Adiposity | Addiction/substance abuse  Depression symptoms | Nil | Body dissatisfaction | Sleep quality | Nil | Dietary restraint  Disinhibition  Food rules | Blood pressure  Blood lipids  Insulin and glucose  Liver enzymes |
| Raman et al., 2018 (52); Raman, Hay & Smith, 2014 (53); Australia; University; January 2013 – March 2014 | Age  Sex/gender  Language status  Partner status  Education status  Employment status | Eating disorder history  Medication history | Nil | Weight  Height  Waist circumference | Mental health diagnoses  Addiction/substance abuse  Depression symptoms  Anxiety symptoms | Nil | Quality of life  Stress | Nil | Nil | Grazing | Nil |
| Raynor et al., 2012 (54); LaRose et al., 2014 (55); USA; University; July 2006 – August 2008 | Age  Sex/gender  Race/ethnicity  Partner status  Education status | Medical history  Medication history | Prior weight loss attempts | Weight  Height | Nil | Nil | Nil | Sleep quality | Nil | Dietary restraint  Disinhibition | Nil |
| Rieger et al., 2017 (56); Rieger et al., 2014 (57); Australia; University & community; May 2010 – March 2012 | Age  Sex/gender  Language status  Partner status  Education status  Employment status  Support | Medical history  Medication history | Nil | Weight  Height  Waist circumference | Depression symptoms  Anxiety symptoms | Nil | Self-esteem  Body dissatisfaction  Quality of life  Stress | Nil | Nil | Self-efficacy | Blood pressure  Blood lipids  C-reactive protein  Insulin and glucose |
| Salvo et al., 2022 (58); Salvo et al., 2018 (59); Brazil; Primary care; NR | Age  Sex/gender  Household income  Partner status  Education status | Medical history  Medication history | Nil | Weight  Height  Waist circumference  Adiposity | Addiction/substance abuse  Depression symptoms  Anxiety symptoms | Nil | Nil | Nil | Nil | Dietary restraint  Emotional eating  External eating | Blood lipids  C-reactive protein  Insulin and glucose |
| Seimon et al. 2019 (60); Seimon et al., 2020 (61); Seimon et al., 2018 (62); Australia; University & virtual; March 2013 – July 2016 | Age  Sex/gender  Race/ethnicity | Medical history  Eating disorder history  Medication history | Nil | Weight  Height  Waist circumference  Adiposity | Depression symptoms  Anxiety symptoms | Personality traits | Self-esteem  Quality of life  Stress | Sleep quality | Nil | Dietary restraint  Disinhibition  Emotional eating  Self-efficacy  External eating | Blood pressure |
| Sherwood et al., 2013 (63); Pacanowski et al., 2014 (64); Sherwood et al., 2011 (65); USA; Virtual (phone); May 2007 – September 2008 | Age  Sex/gender  Race/ethnicity  Household structure  Household income  Partner status  Education status  Employment status  Support | Medical history  Eating disorder history  Life events which may impact upon body weight | Prior weight loss attempts  Type of prior weight loss attempts | Weight  Height | Mental health diagnoses  Addiction/substance abuse  Depression symptoms | Nil | Body dissatisfaction | Nil | Nil | Nil | Nil |
| Smith & Whittingham, 2017 (66); Australia; Community; July 2016 – December 2017 | Age  Sex/gender  Race/ethnicity  Household income  Partner status | Medical history  Medication history | Nil | Weight  Height  Adiposity | Depression symptoms  Anxiety symptoms | Nil | Quality of life  Stress | Nil | Nil | Grazing | Nil |
| Smith et al., 2018 (67); USA; University; September 2006 | Age  Sex/gender  Household income  Partner status  Education status  Employment status | Eating disorder history  Medication history | Nil | Nil^1^ | Depression symptoms  Anxiety symptoms | Personality traits | Stress | Nil | Nil | Dietary restraint  Disinhibition | Nil |
| Whitelock et al., 2019 (68); UK; University; September 2017 – February 2018 | Age  Sex/gender  Race/ethnicity  Education status | Eating disorder history  Medication history | Nil | Weight  Height  Adiposity | Nil | Nil | Nil | Nil | Nil | Dietary restraint  Disinhibition  Emotional eating | Nil |
| Williamson et al., 2008 (69); Anton et al., 2008 (70); Heilbronn et al., 2006 (71); USA; University; March 2002 – August 2004 | Age  Sex/gender  Race/ethnicity | Medical history  Eating disorder history | Nil | Weight  Height  Waist circumference  Adiposity | Mental health diagnoses  Addiction, substance/smoking use  Depression symptoms | Nil | Body dissatisfaction  Quality of life | Nil | Nil | Dietary restraint  Disinhibition  Food rules | Blood pressure  Blood lipids  C-reactive protein  Insulin and glucose  Liver enzymes |

**Abbreviations:** NR = not reported; UK=United Kingdom; USA=United States of America

1 Anthropometry was assessed in the trial, however data could not be provided. As per eligibility criteria, participants had overweight or obesity as reported by the trialist.

**Supplementary Table 2: This table outlines, across both adolescent and adult datasets, the number of datasets which provided data in a domain, as well as the median (range) of factors per dataset for each domain. Factors are potentially relevant to eating disorder risk and have been thematically grouped into the below domains.**

|  | **Adolescents** | | **Adults** | |
| --- | --- | --- | --- | --- |
| **Domains (total number of factors within the domain (n))** | **Number of datasets providing data within the domain^a^** | **Median (range) of factors per dataset** | **Number of datasets providing data within the domain^b^** | **Median (range) of factors per dataset** |
| Demographics (n=14) | 20 | 7 (2-10) | 26 | 6 (2-9) |
| Anthropometry (n=4) | 20 | 3 (2-4) | 25 | 3 (2-4) |
| Medical and family history (n=7) | 17 | 3 (1-5) | 26 | 2 (1-5) |
| History of dieting and weight loss attempts (n=3) | 6 | 3 (2-3) | 7 | 2 (1-3) |
| Mental health (n=6) | 19 | 2 (1-4) | 23 | 2 (1-5) |
| Personality traits (n=1) | 0 | N/A | 3 | 1 (1) |
| Psychosocial health (n=4) | 15 | 2 (1-3) | 17 | 1 (1-4) |
| Sleep quality (n=1) | 2 | 1 (1) | 6 | 1 (1) |
| Weight stigma (n=3) | 4 | 1 (1-2) | 0 | N/A |
| Eating behaviours (n=10) | 10 | 3 (1-4) | 23 | 2 (1-5) |
| Cardiometabolic health (n=5) | 8 | 5 (1-5) | 13 | 4 (1-5) |

^a^ The total number of adolescent datasets is 20

^b^ The total number of adult datasets is 26

**Supplementary File References:**

1. Bean MK, LaRose JG, Raynor HA, Adams EL, Evans RK, Farthing S, et al. The role of parents in adolescent obesity treatment: results of the TEENS+ randomized clinical pilot trial. Pediatr Obes. 2022;17(3):e12858. https://doi.org/10.1111/ijpo.12858

2. Raynor HA, Mazzeo SE, LaRose JG, Adams EL, Thornton LM, Caccavale LJ, Bean MK. Effect of a high-intensity dietary intervention on changes in dietary intake and eating pathology during a multicomponent adolescent obesity intervention. Nutrients. 2021;13(6):1850. https://doi.org/10.3390/nu13061850

3. Bonham MP, Dordevic AL, Ware RS, Brennan L, Truby H. Evaluation of a commercially delivered weight management program for adolescents. J Pediatr. 2017;185:73-80. e3. https://doi.org/10.1016/j.jpeds.2017.01.042

4. Dordevic AL, Bonham MP, Ware RS, Brennan L, Truby H. Study protocol: evaluation of ‘JenMe’, a commercially-delivered weight management program for adolescents: a randomised controlled trial. BMC Public Health. 2015;15:1-8. https://doi.org/10.1186/s12889-015-1923-y

5. Braet C, Tanghe A, Decaluwé V, Moens E, Rosseel Y. Inpatient treatment for children with obesity: weight loss, psychological well-being, and eating behavior. J Pediatr Psychol. 2004;29(7):519-29. 10.1093/jpepsy/jsh054

6. Croker H, Viner RM, Nicholls D, Haroun D, Chadwick P, Edwards C, et al. Family-based behavioural treatment of childhood obesity in a UK National Health Service setting: randomized controlled trial. Int J Obes. 2012;36(1):16-26. https://doi.org/10.1038/ijo.2011.182

7. Doyle AC, Goldschmidt A, Huang C, Winzelberg AJ, Taylor CB, Wilfley DE. Reduction of overweight and eating disorder symptoms via the Internet in adolescents: a randomized controlled trial. J Adolesc Health. 2008;43(2):172-9. https://doi.org/10.1016/j.jadohealth.2008.01.011

8. Jelalian E, Mehlenbeck R, Lloyd-Richardson E, Birmaher V, Wing R. ‘Adventure therapy’ combined with cognitive-behavioral treatment for overweight adolescents. Int J Obes. 2006;30(1):31-9. https://doi.org/10.1038/sj.ijo.0803069

9. Jelalian E, Evans EW, Rancourt D, Ranzenhofer L, Taylor N, Hart C, et al. JOIN for ME: testing a scalable weight control intervention for adolescents. Child Obes. 2020;16(3):192-203. https://doi.org/10.1089/chi.2019.0053

10. Darling KE, Rancourt D, Evans EW, Ranzenhofer LM, Jelalian E. Adolescent weight management intervention in a nonclinical setting: changes in eating-related cognitions and depressive symptoms. J Dev Behav Pediatr. 2021;42(7):579-87. https://doi.org/10.1097/DBP.0000000000000929

11. Lister NB, Baur LA, House ET, Alexander S, Brown J, Collins CE, et al. Intermittent energy restriction for adolescents with obesity: the Fast Track to Health randomized clinical trial. JAMA Pediatr. 2024;178(10):1006-16. https://doi.org/10.1001/jamapediatrics.2024.2869

12. Jebeile H, Baur LA, Kwok C, Alexander S, Brown J, Collins CE, et al. Symptoms of depression, eating disorders, and binge eating in adolescents with obesity: the Fast Track to Health randomized clinical trial. JAMA Pediatr. 2024;178(10):996-1005. https://doi.org/10.1001/jamapediatrics.2024.2851

13. Lister NB, Jebeile H, Truby H, Garnett SP, Varady KA, Cowell CT, et al. Fast track to health - intermittent energy restriction in adolescents with obesity. A randomised controlled trial study protocol. Obes Res Clin Pract. 2020;14(1):80-90. https://doi.org/10.1016/j.orcp.2019.11.005

14. Lofrano-Prado MC, Donato Junior J, Lambertucci AC, Lambertucci RH, Malik N, Ritti-Dias RM, et al. Recreational physical activity improves adherence and dropout in a non-intensive behavioral intervention for adolescents with obesity. Research Quarterly for Exercise and Sport. 2022;93(4):659-69. 10.1080/02701367.2021.1893259

15. Mehlenbeck RS, Jelalian E, Lloyd‐Richardson EE, Hart CN. Effects of behavioral weight control intervention on binge eating symptoms among overweight adolescents. Psychol Sch. 2009;46(8):776-86. https://doi.org/10.1002/pits.20416

16. Jelalian E, Lloyd-Richardson EE, Mehlenbeck RS, Hart CN, Flynn-O'Brien K, Kaplan J, et al. Behavioral weight control treatment with supervised exercise or peer-enhanced adventure for overweight adolescents. J Pediatr. 2010;157(6):923-8. e1. https://doi.org/10.1016/j.jpeds.2010.05.047

17. Newsome FA, Cardel MI, Chi X, Lee AM, Miller D, Menon S, et al. Wellness achieved through changing habits: a randomized controlled trial of an acceptance-based intervention for adolescent girls with overweight or obesity. Child Obes. 2023;19(8):525-34. https://doi.org/10.1089/chi.2022.0116

18. Ramalho S, Saint-Maurice PF, Silva D, Mansilha HF, Silva C, Gonçalves S, et al. APOLO-Teens, a web-based intervention for treatment-seeking adolescents with overweight or obesity: study protocol and baseline characterization of a Portuguese sample. Eat Weight Disord. 2020;25:453-63. https://doi.org/10.1007/s40519-018-0623-x

19. Skjåkødegård HF, Conlon RPK, Hystad SW, Roelants M, Olsson SJG, Frisk B, et al. Family-based treatment of children with severe obesity in a public healthcare setting: results from a randomized controlled trial. Clin Obes. 2022;12(3):e12513. https://doi.org/10.1111/cob.12513

20. Skjåkødegård HF, Danielsen YS, Morken M, Linde S-RF, Kolko RP, Balantekin KN, et al. Study protocol: a randomized controlled trial evaluating the effect of family-based behavioral treatment of childhood and adolescent obesity–The FABO-study. BMC Public Health. 2016;16:1-9. https://doi.org/10.1186/s12889-016-3755-9

21. Vermeiren E, Naets T, Van Eyck A, Vervoort L, Ysebaert M, Baeck N, et al. Improving treatment outcome in children with obesity by an online self-control training: a randomized controlled trial. Front Pediatr. 2021;9. https://doi.org/10.3389/fped.2021.794256

22. Naets T, Vervoort L, Ysebaert M, Van Eyck A, Verhulst S, Bruyndonckx L, et al. WELCOME: improving WEight controL and CO-Morbidities in children with obesity via Executive function training: study protocol for a randomized controlled trial. BMC Public Health. 2018;18:1-10. https://doi.org/10.1186/s12889-018-5950-3

23. Vidmar AP, Naguib M, Raymond JK, Salvy SJ, Hegedus E, Wee CP, Goran MI. Time-limited eating and continuous glucose monitoring in adolescents with obesity: a pilot study. Nutrients. 2021;13(11):3697. https://doi.org/10.3390/nu13113697

24. Vidmar AP, Goran MI, Naguib M, Fink C, Wee CP, Hegedus E, et al. Time limited eating in adolescents with obesity (time LEAd): study protocol. Contemp Clin Trials. 2020;95:106082. https://doi.org/10.1016/j.cct.2020.106082

25. Vidmar AP, Salvy SJ, Wee CP, Pretlow R, Fox DS, Yee JK, et al. An addiction‐based digital weight loss intervention: a multi‐centre randomized controlled trial. Pediatr Obes. 2023;18(3):e12990. https://doi.org/10.1111/ijpo.12990

26. Vidmar AP, Salvy SJ, Pretlow R, Mittelman SD, Wee CP, Fink C, et al. An addiction-based mobile health weight loss intervention: protocol of a randomized controlled trial. Contemporary Clinical Trials. 2019;78:11-9. 10.1016/j.cct.2019.01.008

27. Epstein LH, Paluch RA, Saelens BE, Ernst MM, Wilfley DE. Changes in eating disorder symptoms with pediatric obesity treatment. J Pediatr. 2001;139(1):58-65. https://doi.org/10.1067/mpd.2001.115022

28. Shomaker LB, Tanofsky‐Kraff M, Matherne CE, Mehari RD, Olsen CH, Marwitz SE, et al. A randomized, comparative pilot trial of family‐based interpersonal psychotherapy for reducing psychosocial symptoms, disordered‐eating, and excess weight gain in at‐risk preadolescents with loss‐of‐control‐eating. Int J Eat Disord. 2017;50(9):1084-94. https://doi.org/10.1002/eat.22741

29. Wilfley DE, Stein RI, Saelens BE, Mockus DS, Matt GE, Hayden-Wade HA, et al. Efficacy of maintenance treatment approaches for childhood overweight: a randomized controlled trial. JAMA. 2007;298(14):1661-73. https://doi.org/10.1001/jama.298.14.1661

30. Goldschmidt AB, Best JR, Stein RI, Saelens BE, Epstein LH, Wilfley DE. Predictors of child weight loss and maintenance among family-based treatment completers. J Consult Clin Psychol. 2014;82(6):1140. https://doi.org/10.1037/a0037169

31. Wilfley DE, Saelens BE, Stein RI, Best JR, Kolko RP, Schechtman KB, et al. Dose, content, and mediators of family-based treatment for childhood obesity: a multisite randomized clinical trial. JAMA Pediatr. 2017;171(12):1151-9. https://doi.org/10.1001/jamapediatrics.2017.2960

32. Barnes RD, White MA, Martino S, Grilo CM. A randomized controlled trial comparing scalable weight loss treatments in primary care. Obesity. 2014;22(12):2508-16. https://doi.org/10.1002/oby.20889

33. Barnes RD, Ivezaj V, Martino S, Pittman BP, Grilo CM. Back to basics? No weight loss from motivational interviewing compared to nutrition psychoeducation at one‐year follow‐up. Obesity. 2017;25(12):2074-8. https://doi.org/10.1002/oby.21972

34. Beaulieu K, Casanova N, Oustric P, Turicchi J, Gibbons C, Hopkins M, et al. Matched weight loss through intermittent or continuous energy restriction does not lead to compensatory increases in appetite and eating behavior in a randomized controlled trial in women with overweight and obesity. The Journal of nutrition. 2020;150(3):623-33. 10.1093/jn/nxz296

35. Boutelle KN, Eichen DM, Peterson CB, Strong DR, Kang-Sim D-JE, Rock CL, Marcus BH. Effect of a novel intervention targeting appetitive traits on body mass index among adults with overweight or obesity: a randomized clinical trial. JAMA Netw Open. 2022;5(5):e2212354-e. https://doi.org/10.1001/jamanetworkopen.2022.12354

36. Boutelle KN, Pasquale EK, Strong DR, Eichen DM, Peterson CB. Reduction in eating disorder symptoms among adults in different weight loss interventions. Eat Behav. 2023;51:101787. https://doi.org/10.1016/j.eatbeh.2023.101787

37. Boutelle KN, Eichen DM, Peterson CB, Strong DR, Rock CL, Marcus BH. Design of the PACIFIC study: a randomized controlled trial evaluating a novel treatment for adults with overweight and obesity. Contemp Clin Trials. 2019;84:105824. https://doi.org/10.1016/j.cct.2019.105824

38. Carpenter KM, Vickerman KA, Salmon EE, Javitz HS, Epel ES, Lovejoy JC. A randomized pilot study of a phone-based mindfulness and weight loss program. Behav Med. 2019;45(4):271-81. https://doi.org/10.1080/08964289.2017.1384359

39. Dalle Grave R, Calugi S, Gavasso I, El Ghoch M, Marchesini G. A randomized trial of energy‐restricted high‐protein versus high‐carbohydrate, low‐fat diet in morbid obesity. Obesity. 2013;21(9):1774-81. https://doi.org/10.1002/oby.20320

40. Dassen FC, Houben K, Van Breukelen GJ, Jansen A. Gamified working memory training in overweight individuals reduces food intake but not body weight. Appetite. 2018;124:89-98. https://doi.org/10.1016/j.appet.2017.05.009

41. Eichen DM. Novel Executive Function Training for Obesity Study Protocol [PDF] 2021 [updated 12/07/2021. Available from: https://cdn.clinicaltrials.gov/large-docs/96/NCT03724396/Prot_SAP_000.pdf.

42. Griffin H, Cheng H, O'Connor H, Rooney K, Petocz P, Steinbeck K. Higher protein diet for weight management in young overweight women: a 12‐month randomized controlled trial. Diabetes Obes Metab. 2013;15(6):572-5. https://doi.org/10.1111/dom.12056

43. Cheng HL, Griffin H, Claes B-E, Petocz P, Steinbeck K, Rooney K, O’Connor H. Influence of dietary macronutrient composition on eating behaviour and self-perception in young women undergoing weight management. Eat Weight Disord. 2014;19:241-7. https://doi.org/10.1007/s40519-014-0110-y

44. Imayama I, Alfano CM, Kong A, Foster-Schubert KE, Bain CE, Xiao L, et al. Dietary weight loss and exercise interventions effects on quality of life in overweight/obese postmenopausal women: a randomized controlled trial. Int J Behav Nutr Phys Act. 2011;8:1-12. https://doi.org/10.1186/1479-5868-8-118

45. Mason C, de Dieu Tapsoba J, Duggan C, Wang C-Y, Alfano CM, McTiernan A. Eating behaviors and weight loss outcomes in a 12-month randomized trial of diet and/or exercise intervention in postmenopausal women. Int J Behav Nutr Phys Act. 2019;16:1-11. https://doi.org/10.1186/s12966-019-0887-1

46. Jospe MR, Roy M, Brown RC, Williams SM, Osborne HR, Meredith‐Jones KA, et al. The effect of different types of monitoring strategies on weight loss: a randomized controlled trial. Obesity. 2017;25(9):1490-8. https://doi.org/10.1002/oby.21898

47. Jospe MR, Brown RC, Williams S, Roy M, Meredith‐Jones K, Taylor R. Self‐monitoring has no adverse effect on disordered eating in adults seeking treatment for obesity. Obes Sci Pract. 2018;4(3):283-8. https://doi.org/10.1002/osp4.168

48. Taylor RW, Roy M, Jospe MR, Osborne HR, Meredith-Jones KJ, Williams SM, Brown RC. Determining how best to support overweight adults to adhere to lifestyle change: protocol for the SWIFT study. BMC Public Health. 2015;15:1-11. https://doi.org/10.1186/s12889-015-2205-4

49. Lin S, Cienfuegos S, Ezpeleta M, Gabel K, Pavlou V, Mulas A, et al. Time-restricted eating without calorie counting for weight loss in a racially diverse population: a randomized controlled trial. Ann Intern Med. 2023;176(7):885-95. https://doi.org/10.7326/M23-0052

50. Martin CK, Johnson WD, Myers CA, Apolzan JW, Earnest CP, Thomas DM, et al. Effect of different doses of supervised exercise on food intake, metabolism, and non-exercise physical activity: the E-MECHANIC randomized controlled trial. Am J Clin Nutr. 2019;110(3):583-92. https://doi.org/10.1093/ajcn/nqz054

51. Myers CA, Johnson WD, Earnest CP, Rood JC, Tudor-Locke C, Johannsen NM, et al. Examination of mechanisms (E-MECHANIC) of exercise-induced weight compensation: study protocol for a randomized controlled trial. Trials. 2014;15:1-12. https://doi.org/10.1186/1745-6215-15-212

52. Raman J, Hay P, Tchanturia K, Smith E. A randomised controlled trial of manualized cognitive remediation therapy in adult obesity. Appetite. 2018;123:269-79. 10.1016/j.appet.2017.12.023

53. Raman J, Hay P, Smith E. Manualised Cognitive Remediation Therapy for adult obesity: study protocol for a randomised controlled trial. Trials. 2014;15:1-9. https://doi.org/10.1186/1745-6215-15-426

54. Raynor HA, Steeves EA, Hecht J, Fava JL, Wing RR. Limiting variety in non-nutrient-dense, energy-dense foods during a lifestyle intervention: a randomized controlled trial. Am J Clin Nutr. 2012;95(6):1305-14. https://doi.org/10.3945/ajcn.111.031153

55. LaRose JG, Fava JL, Steeves EA, Hecht J, Wing RR, Raynor HA. Daily self-weighing within a lifestyle intervention: impact on disordered eating symptoms. Health Psychol. 2014;33(3):297. https://doi.org/10.1037/a0034218

56. Rieger E, Treasure J, Murray K, Caterson I. The use of support people to improve the weight-related and psychological outcomes of adults with obesity: A randomised controlled trial. Behaviour Research and Therapy. 2017;94:48-59. 10.1016/j.brat.2017.04.012

57. Rieger E, Treasure J, Swinbourne J, Adam B, Manns C, Caterson I. The effectiveness of including support people in a cognitive behavioural weight loss maintenance programme for obese adults: study rationale and design. Clin Obes. 2014;4(2):77-90. https://doi.org/10.1111/cob.12042

58. Salvo V, Curado DF, Sanudo A, Kristeller J, Schveitzer MC, Favarato ML, et al. Comparative effectiveness of mindfulness and mindful eating programmes among low-income overweight women in primary health care: A randomised controlled pragmatic study with psychological, biochemical, and anthropometric outcomes. Appetite. 2022;177:106131. 10.1016/j.appet.2022.106131

59. Salvo V, Kristeller J, Montero Marin J, Sanudo A, Lourenço BH, Schveitzer MC, et al. Mindfulness as a complementary intervention in the treatment of overweight and obesity in primary health care: study protocol for a randomised controlled trial. Trials. 2018;19(1):277. https://doi.org/10.1186/s13063-018-2639-y

60. Seimon RV, Wild-Taylor AL, Keating SE, McClintock S, Harper C, Gibson AA, et al. Effect of weight loss via severe vs moderate energy restriction on lean mass and body composition among postmenopausal women with obesity: the TEMPO diet randomized clinical trial. JAMA Netw Open. 2019;2(10):e1913733-e. https://doi.org/10.1001/jamanetworkopen.2019.13733

61. Seimon RV, Wild-Taylor AL, McClintock S, Harper C, Gibson AA, Johnson NA, et al. 3-Year effect of weight loss via severe versus moderate energy restriction on body composition among postmenopausal women with obesity-the TEMPO Diet Trial. Heliyon. 2020;6(6). https://doi.org/10.1016/j.heliyon.2020.e04007

62. Seimon RV, Gibson AA, Harper C, Keating SE, Johnson NA, Da Luz FQ, et al. Rationale and protocol for a randomized controlled trial comparing fast versus slow weight loss in postmenopausal women with obesity—the TEMPO Diet Trial. Healthcare. 2018;6(3):85. https://doi.org/10.3390/healthcare6030085

63. Sherwood NE, Crain AL, Martinson BC, Anderson CP, Hayes MG, Anderson JD, et al. Enhancing long-term weight loss maintenance: two-year results from the Keep It Off randomised controlled trial. Prev Med. 2013;56(3-4):171-7. https://doi.org/10.1016/j.ypmed.2012.12.014

64. Pacanowski CR, Senso MM, Oriogun K, Crain AL, Sherwood NE. Binge eating behavior and weight loss maintenance over a 2‐year period. J Obes. 2014;2014(1):249315. https://doi.org/10.1155/2014/249315

65. Sherwood NE, Crain AL, Martinson BC, Hayes MG, Anderson JD, Clausen JM, et al. Keep it off: a phone-based intervention for long-term weight-loss maintenance. Contemp Clin Trials. 2011;32(4):551-60. https://doi.org/10.1016/j.cct.2011.03.011

66. Smith E, Whittingham C. Cognitive remediation therapy plus behavioural weight loss compared to behavioural weight loss alone for obesity: study protocol for a randomised controlled trial. Trials. 2017;18:1-7. https://doi.org/10.1186/s13063-017-1778-x

67. Smith BW, Shelley BM, Sloan AL, Colleran K, Erickson K. A preliminary randomized controlled trial of a mindful eating intervention for post-menopausal obese women. Mindfulness. 2018;9:836-49. https://doi.org/10.1007/s12671-017-0824-9

68. Whitelock V, Kersbergen I, Higgs S, Aveyard P, Halford JC, Robinson E. A smartphone based attentive eating intervention for energy intake and weight loss: results from a randomised controlled trial. BMC Public Health. 2019;19:1-11. https://doi.org/10.1186/s12889-019-6923-x

69. Williamson DA, Martin CK, Anton SD, York-Crowe E, Han H, Redman L, Ravussin E. Is caloric restriction associated with development of eating-disorder symptoms? Results from the CALERIE trial. Health Psychol. 2008;27(1S):S32. https://doi.org/10.1037/0278-6133.27.1.S32

70. Anton S, Martin C, Redman L, York-Crowe E, Heilbronn L, Han H, et al. Psychosocial and behavioral pre-treatment predictors of weight loss outcomes. Eat Weight Disord. 2008;13:30-7. https://doi.org/10.1007/BF03327782

71. Heilbronn LK, De Jonge L, Frisard MI, DeLany JP, Larson-Meyer DE, Rood J, et al. Effect of 6-month calorie restriction on biomarkers of longevity, metabolic adaptation, and oxidative stress in overweight individuals: a randomized controlled trial. JAMA. 2006;295(13):1539-48. https://doi.org/10.1001/jama.295.13.1539.
